# The Acceptability of Online Consent in a Self-Test Serosurvey of Responders to the 2014–2016 West African Ebola Outbreak

**DOI:** 10.1093/phe/phx027

**Published:** 2017-12-22

**Authors:** Catherine R McGowan, Catherine F Houlihan, Patricia Kingori, Judith R Glynn

**Affiliations:** 1 Faculty of Public Health and Policy, London School of Hygiene and Tropical Medicine and Humanitarian Public Health Technical Unit, Save the Children; 2 Faculty of Infectious and Tropical Diseases, London School of Hygiene and Tropical Medicine and Faculty of Medical Sciences, University College London; 3 Ethox Centre, Nuffield Department of Population Health, University of Oxford; 4 Faculty of Epidemiology and Population Health, London School of Hygiene and Tropical Medicine

## Abstract

Online participation in research is used increasingly to recruit geographically dispersed populations. Obtaining online consent is convenient, yet we know little about the acceptability of this practice. We carried out a serostudy among personnel returning to the UK/Ireland following deployment to West Africa during the 2014–2016 Ebola epidemic. We used an online procedure for consenting returnees and designed a small descriptive study to understand: how much of the consent material they read, how informed they felt and if they preferred online to traditional face-to-face consent. Of 261 returnees, 111 (43 per cent) completed the consent survey. Participants indicated a high level of engagement with the consent materials, with 67 per cent reporting having read all and 20 per cent having read ‘most’ of the materials. All participants indicated feeling completely (78 per cent) or mostly (22 per cent) informed about the purpose, methods and intended uses of the research, as well as what participation was required and what risks were involved. Only three participants indicated a preference for face-to-face consent. Free-text comments suggested that online consent may be an acceptable modality for uncomplicated and low-risk studies. The study sample was largely composed of health professionals, suggesting acceptability of online consent within this population.

## Introduction

Informed consent is the process whereby participants are provided with information about a research study and agree to participate based on mutually acceptable terms. Informed consent is based on complete information regarding the purpose, process, benefits and potential harms of the research and requires that participants have the capacity to make a fully autonomous, reasoned decision to participate. It has usually been obtained from face-to-face interaction with a researcher present to provide the requisite information about the study, to respond to queries, ensure understanding, assess capacity to consent and to obtain a signed consent form. Some of the challenges to obtaining informed consent under these conditions include: travel, workflow, scheduling, misgivings about the research topic and difficulties understanding consent materials (Welch *et al.*, 2016). Obtaining consent by non-traditional means may address some of these challenges. Alternatives to face-to-face consent include consent materials posted to participants, ‘teleconsent’, consent built into mobile applications and eConsent (i.e. electronic/online consent). Posting consent forms can be time-consuming and expensive, and places the responsibility for signing and returning materials on the participant, which may reduce response rates. Loss or misdirection of posted materials containing the participant’s name and other personal details presents a potential breach of confidentiality and distress to participants. To illustrate this point, in 2015/2016 Royal Mail reported successful delivery of 99.7 per cent of posted items (The Office of Communications 2016). Assuming consent forms are posted to and from participants, this translates to six missing consent forms for a survey of 1000 participants.

‘Teleconsent’ has been proposed as a means of providing online consent for clinical trials with the participant and a member of the research team carrying out the consent process together in real time (Welch *et al.*, 2016). Increasingly, bespoke mobile applications are used to facilitate real-time data collection from participants, and can also be used to obtain consent (Doerr *et al.*, 2017). However, these alternative approaches are not necessarily regarded as an improvement. Recently, the app-mediated Parkinson mPower study reported that its application fared no better than obtaining consent face-to-face: ‘[d]espite attention to presentation, content flow, and the use of icons, animations, and video as well as the volume of the information presented, we identified broad thematic consistency with gross challenges observed in in-person, fully facilitated informed consent processes’ (Doerr *et al.*, 2017: 9).

Electronic consent requires participants to access Web-based consent materials and to indicate consent online. In a survey of 750 University Human Research Ethics Boards in the USA, Research Ethics Committee (REC) members raised issues about the lack of formal consent when data were collected by online surveys, with one REC member drawing attention to the fact that, ‘[t]here still seems to be a widespread misconception that there is such a thing as implied consent or passive consent[;] that is, if you fill the survey out, you are consenting to doing it…’ (Buchanan and Hvizdak, 2009, p. 43). Thus, the suggestion to strengthen the ethical conduct of online surveys is to ensure that the consent process is more explicit and formal. One means to do this is to embed the consent documents within the online survey; however, this process is not without its challenges.

In a survey of UK-based researchers, concerns were raised about eConsent, including the limitations it puts on the researcher’s ability to assess capacity to consent (Stevens *et al.*, 2016). In the survey of 95 researchers, only one reported having used using both online consent materials and online patient information sheets. Eighty-seven (92 per cent) researchers reported never having used either online consent or online patient information sheets; however, 84 per cent believed that UK regulators would allow eConsent for studies where the risk to participants was minimal (Stevens *et al.*, 2016).

Though there is some published literature presenting the views of ethics committee members about online consent (Buchanan and Hvizdak, 2009), as well as the concerns of UK-based researchers (Stevens *et al.*, 2016), there are few studies presenting the viewpoint of research participants. An unpublished pilot of a consent and data management system surveyed 21 patients: 12 participants reported being satisfied with using the e-platform, though various concerns were raised (Collins *et al.*, 2015). Patients indicated a lack of clarity regarding what they were consenting to; some were unable to distinguish between the various types of consent they were asked to provide. Others were unclear about the implications of consent. The research team concluded that, ‘[w]hile study participants are inclined to use e-platforms and researchers use electronic methods to collect data (tablets, etc.); executing eConsent remains a challenge’ (Collins *et al.*, 2015).

A 1997 study looking at the use of eConsent among endoscopy patients concluded that, ‘…patient satisfaction should be a factor in determining the best method of providing informed consent information’ (Agre *et al.*, 1997: 162). Similarly, a 2016 study comparing the institutional review board (IRB) professionals’ views on consent to those of patients determined that patient preferences for consent often differ from that of IRBs and highlighted the need to, ‘integrate patient preferences into prevailing regulatory interpretations’ (Kraft *et al.*, 2016: 555).

Recently, the US Department of Health and Human Services published guidelines on the use of electronic informed consent (US Department of Health and Human Services *et al.*, 2016). The guidelines seek to provide practical recommendations for the use of online consent; however, they do not provide insight into the acceptability of online consent beyond indicating that, ‘[…]subjects should have the option to use paper-based or electronic informed consent methods completely or partially throughout the informed consent process’, and that ‘some subjects may prefer one method over another’ (US Department of Health and Human Services *et al.*, 2016: 4).

This paper presents the results of a survey of 111 research participants regarding the acceptability of online consent, conducted among international responders to the West African Ebola outbreak.

## Background

We undertook a study to determine the prevalence of past sub-clinical and asymptomatic infection with Ebola virus in returning responders. The study was administered using a questionnaire to record possible exposures and symptoms (using an online survey created using Bristol Online Surveys (University of Bristol, 2017)) and required the collection of an oral fluid sample for testing for Ebola virus antibodies (Houlihan *et al.*, 2017). Responders were dispersed geographically throughout the UK and Ireland. The survey requested a mailing address to which we posted an oral self-test kit with instructions on the collection and handling of the sample. Participants returned samples via regular post, in a pre-paid envelope.

While designing the study we considered several options for obtaining consent from participants. It was possible that some participants would have evidence of past infection – and all participants were potentially subject to stigma owing to their participation in the Ebola response and potential exposure to a virus that had inspired a considerable degree of social paranoia – as such, confidentiality and anonymity were particularly important.

Face-to-face consent was not practical given the geographical distribution of responders. We considered posting consent forms but opted not to use this method owing to the inconvenience for potential participants (who would need to post both the consent form and subsequently the oral sample), but also because both the information sheet and the consent form referenced deployment to West Africa as part of the Ebola response, and potential antibody seropositivity (indicating past exposure to or previous infection with Ebola virus). As these forms would be posted to named individuals, we felt there was a risk of exposing participants as having potentially been exposed to a highly stigmatized disease should the post be delivered to, or opened by, someone other than the intended recipient.

We therefore used an online consent procedure embedded at the beginning of the online questionnaire (Appendix 1), and were granted ethics approval for the study, including the use of online consent, from the London School of Hygiene and Tropical Medicine (LSHTM) Research Ethics Committee (Approval reference 9475). The online consent materials consisted of an information sheet describing the purpose of the study, what participation involved and a description of the participants’ rights. The materials also included a series of statements clarifying what participants would be consenting to, along with the contact details of two of the investigators and the Chair of the Research Ethics Committee at the LSHTM.

Participants who did not indicate consent were not able to access the online questionnaire (conditional routing of the survey tool would decline access to the survey to those who had not indicated consent), nor could they provide a mailing address to which we could send the self-test materials. Instead they were routed to a ‘screened out’ message. A challenge of this design was that we were unable to determine how many individuals had declined to consent and were thus screened out.

Participants were given the option to receive their results via email, post or telephone at the end of the study. The two participants who tested positive for antibodies to Ebola virus requested notification by email and were informed using this method initially (by the primary investigator) before follow-up telephone contact was made (Houlihan *et al.*, 2017). A link to a short online survey asking participants about the online consent procedure was included in the email notification sent to those with a negative test result.

## Methods

The online consent survey was created using Bristol Online Surveys and asked participants to indicate: how much of the consent material they recalled reading, to what degree they felt informed about the study, and how they felt about online compared to face-to-face consent. If a participant indicted that they did not feel fully informed they were routed to a question asking them to describe what they felt uninformed about; they were also asked if they would have felt more informed had the consent procedure been carried out face-to-face. A free-text field was included at the end of the survey to allow participants to comment on the original consent process, or to provide clarification on any of their survey responses. The survey was anonymous. As this survey falls under the definition of service evaluation, approval from the LSHTM REC was not required.

## Results

Of the 268 returnees who submitted a sample, 261 were notified of their test result via email. Two tested positive for antibodies and were thus approached separately by the primary investigator, four requested to be notified of their test result by phone/post and one provided an invalid email address and could not be contacted. Returnees included both clinical and non-clinical staff.

Of the 261 returnees who were sent an email including the link to the online consent survey, 111 (43 per cent) completed the survey. As the survey was anonymous, we cannot compare characteristics of those who did or did not respond. We did not ask participants in the consent survey to indicate their occupational role; however, returnees provided this information as part of the original antibody study. The sampling frame for the consent survey included: laboratory staff (*n* = 95), physicians (*n* = 70), nurses (*n* = 54), researchers (*n* = 37), management/operations (*n* = 28), trainers (*n* = 23), epidemiologists (*n* = 19), community engagement/tracing (*n* = 18), water, sanitation, and hygiene (WASH) (*n* = 11), finance (*n* = 3), engineers (*n* = 3), pharmacists (*n* = 2) and ‘other’ (*n* = 7). Returnees were allowed to select more than one role. As the consent survey sampled from this population, we believe it reasonable to conclude that it largely represents the views of health professionals.

**Figure 1.**
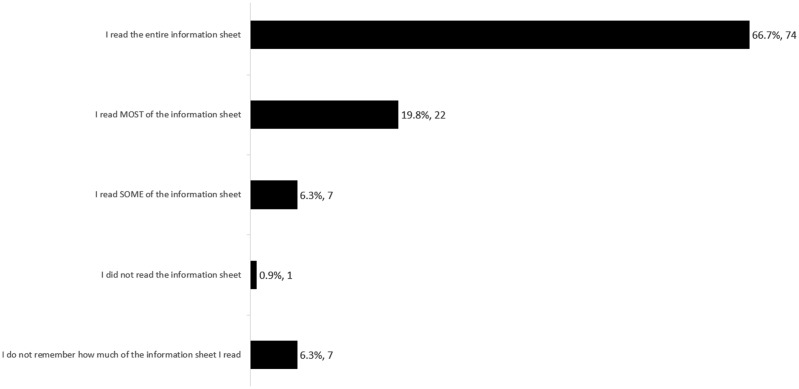
How much of the consent material do you recall reading?

The consent survey asked participants to indicate how much of the online consent material they recall reading (Figure 1). Of the 104 participants who could recall how much of the information sheet they had read, 74 (71 per cent) reported having read *all* and 22 (21 per cent) reported reading *most* of it. Seven participants (7 per cent) reported having read only *some* of the information sheet, while only one (1 per cent) indicated having not read any of it.

Participants were asked to indicate to what degree they felt informed about the purpose, methods and intended uses of the research, as well as what participation was required and what risks, if any, were involved. Participants reported that they felt *completely informed* (78 per cent, *n* = 87) or *mostly informed* (22 per cent, *n* = 24), including those who had read little of the information sheet. None of the participants indicated feeling completely or mostly uninformed. The frequency distribution of the degree to which respondents reported feeling informed is cross tabulated with the reported engagement with the consent materials in Table 1.

**Table 1.** Feeling informed by degree of engagement

Informed	Read all info	Most info	Some info	None	Cannot remember	Total
Completely	64	15	3	0	5	87
Mostly	10	7	4	1	2	24

Participants who felt *mostly informed* were asked to indicate what they did not feel informed about, and whether they thought they would have felt more informed had consent been taken face-to-face. The responses are summarized in Table 2: seven would have felt more informed with face-to-face consent. However, overall, only three (3 per cent) participants indicated that they would have preferred a face-to-face consent procedure to the online consent procedure used for this study.

Overall, fifty-seven participants (52 per cent) indicated a preference for the online consent procedure, while 50 participants (46 per cent) indicated no preference. Two of the three participants who indicated a preference for a face-to-face consent procedure also reported reading all the consent materials and feeling completely informed.

**Table 2.** Those who felt *mostly* informed (*n* = 24)

How much read	Do not think more informed if F2F	Yes, would have felt more informed if F2F	Unsure	What did you feel uninformed about?
Entire sheet (*n* = 10)	5	2	3	As a physician and clinical researcher, I would have been interested (just out of curiosity) to know specific details about the assay used, and any preliminary findings—but those do not really affect the process or appropriateness of consent
				If the sample was to be destroyed afterward or archived*
				Who owned the information, and where it was to be published? (If I remember correctly!)
				I think there were some points which I want to understand more about and some of the technical language and points it would have helped to have been able to ask questions about or have some sort of contact information, e.g. email this address with any questions, etc.* I think it would also be good to hear about the results of the study maybe the results will be presented somewhere and participants in the study could attend. The write-up could be emailed out or information on where it can be found. An opportunity to ask questions about the study would also be great and to hear what others think about the study and its findings
				I think that this approach was OK for a straightforward survey of this kind but not for anything more complicated
				Did not particularly feel uninformed about anything, I just felt I had to read it over again in case I was missing anything
Most (*n* = 7)	1	4	2	I would have liked to have known a lot more about the study
				Probably my fault for skimming the consent form quickly, as I was busy, but wanted to help. I cannot recall feeling informed about what would happen if the result was positive, e.g. would there be any restrictions on my working practices?
				But if I wanted more info I could have got it easily
Some (*n* = 4)	3	1	0	As I did not plow through all the documents not sure what I missed. It was a conscious decision and one I am comfortable with
None (*n* = 1)	1	0	0	
Cannot remember (*n* = 2)	1	0	1	I cannot really remember it was that long ago. By the time I received the sampling kit I had completely forgotten I had signed up until I had opened it

* This information was included in the consent materials (see Appendix 1).

### Comments regarding consent

Participants were also given the opportunity to submit additional comments. Comments fell into two categories: that consent was appropriate for this study but not for something more complex/risker and the convenience of online consent.

#### Consent appropriate for this study but not for a more complex/riskier study

Participants suggested that their preference for the online process was contingent on: the topic, the type of research taking place, the levels of personal risk associated with it and the extent to which it was deemed complex or straightforward with more complex studies being seen to be less appropriate for online consent. Participants also suggested ways that the online consent process could be improved by, for example, making it more interactive with video links between researchers and participants.

**I have said that I have no preference in relation to online versus face to face consent, however, it would depend on the type of trial. A complex treatment trial does require the reassurance for both the participant and the researcher that the participant truly understands the trial and can provide confirmed informed consent. However, a combination of video linking between the researcher and patient with the consent then provided online would solve that issue. Such a system would be very helpful in remote areas (including the Highlands in Scotland) improving recruitment and equity of access for remote and rural patients.**

Participant 50: Reported reading all of the consent materials, felt completely informed and had no preference regarding consent.

**For this type of study online consent was totally appropriate. For a more complicated study face to face would give an opportunity to ask questions. I preferred online for this but in another scenario I may prefer face to face.**

Participant 70: Reported reading all of the consent materials, felt completely informed and preferred online consent over face-to-face.

**I think consent for studies such as this with miniscule risk for adverse outcome to the individual has got way out of hand and a very simple process should be used. Online consent is more than adequate in my opinion. People can after all decline or pick up the phone if they are uncertain.**

Participant 82: Reported not being able to remember how much of the consent materials she/he read, felt completely informed and preferred online consent over face-to-face.

#### Convenience of online consent

Participants suggested that the convenience of being able to consent online meant that they were able to take part in the research suggesting a benefit both to participants (as they were able to participate), and to the study team (as this increased recruitment).

**I probably wouldn't have got round to a face to face interview due to time pressures etc, an online consent form is more practical, with the opportunity to ask questions if necessary. I expected the result to be negative, so wasn't unduly concerned about consent.**

Participant 48: Reported reading most of the consent materials, felt mostly informed and preferred online consent over face-to-face.

**If it improves the efficiency of studies and saves money I can't see why it isn't the standard way these days. As long as people can phone/email someone if they want extra information. Great for studies on people from a wide geographical area like this one.**

Participant 47: Reported reading all of the consent materials, felt completely informed and preferred online consent over face-to-face.

**Although I prefer online consent because of the problem of time tabling involved in face to face consent, I do realise that the temptation exists not to read the information carefully but still ‘consent’ and also the opportunity to ask question if something is unclear is removed when using online consent.**

Participant 15: Reported reading all of the consent materials, felt mostly informed and preferred online consent over face-to-face.

**Efficiently planned and executed. Online consent, with the option to contact someone if desired is entirely appropriate for this community of participants, and beyond.**

Participant 108: Reported reading all of the consent materials, felt completely informed and preferred online consent over face-to-face.

Other benefits reported by participants included being able to read through the online consent materials at their own pace. For example, one participant who reported reading all the materials alluded to having to read the information more than once, ‘**I just felt I had to read it over again in case I was missing anything**’ (Participant 107: Reported reading all of the consent materials, felt mostly informed and had no preference for online consent over face-to-face).

In addition to outlining what they perceived to be some of the advantages of online consent compared to the traditional face-to-face method, participants also commented on what they felt were some of the challenges. For example, some of the above quotes suggest that there was a temptation for participants using online consent not to read the information carefully. Others suggest that this approach to consent would work better with opportunities to obtain additional information from the researchers themselves.

## Discussion

Self-reported engagement with the online consent materials was high, with 87 per cent (*n* = 96) of participants indicating they read *all* or *most* of the materials. We could find no published studies reporting how much of the consent materials research participants read and are therefore not able to determine if this is typical. It seems plausible, however, that participants are more likely to read all the materials, and potentially to gain a higher level of understanding of their contents, when not subject to social pressure to read quickly.

While most participants reported feeling completely informed about the purpose, methods and intended uses of the research, as well as what participation was required and what risks, if any, were involved, some of them had not read all the materials. Further, some of the participants who did not read the consent materials, or who read only some of the materials, indicated feeling completely informed. This discrepancy between the perceptions of being informed and actually having read the consent materials raises important questions about the extent to which valid consent was achieved using this method.

There are a number of possible explanations for this discrepancy. The most probable explanation could be that the participants discussed the study with other potential participants prior to gaining and reading the information from the study team and receiving the online consent materials. We used a snowball sampling approach, where existing study participants were used to recruit future participants from among their acquaintances, and it is possible that during this process the study was discussed. It is also possible that given the demographics of the study population—most were educated professionals—they would feel more pressure than other groups to say that they feel completely informed. As the antibody study employed a novel method (for obtaining and testing oral samples), participants could not have reasonably presumed to feel informed about the study owing to a familiarity with our methodology. Finally, it is possible that there was a point in the consent materials at which people felt satisfied that the benefits of the study outweighed any potential harms and that, on this basis alone, they reported feeling fully informed.

Participants who felt *mostly informed* were asked to indicate what they felt uninformed about. Only two participants mentioned something that was already clearly indicated in the consent materials (i.e. one participant did not believe she/he had been provided with the contact information of someone who could answer questions about the study, and another queried the arrangements for archiving the samples), suggesting generally good comprehension and retention among participants. Research participants are less likely to read long, or overly detailed consent materials; therefore, augmenting online consent forms to include more information to lessen the likelihood of participants feeling uniformed is unlikely to benefit engagement (Antoniou *et al.*, 2011). However, the use of an online platform that allows participants to control the amount of information they access and the option to expand components of the consent materials could have been included. None of the research participants contacted the study team to seek clarification or to request more information, despite the fact that contact details were conspicuously included in the consent materials. The online survey platform we used did not allow the consent materials to be easily downloaded nor did we include the option to have them emailed to participants. The option to download or email consent materials may have reduced the number of participants who did not feel completely informed as this would have allowed them access to the study information, as well as the contact details of the study team, after completing the questionnaire.

A considerable number of participants enquired about the study results, often requesting that the published results be sent to them; this is consistent with other studies which have shown that participants often query the dissemination of study results (Antoniou *et al.*, 2011). Though we contacted each participant to communicate their test results, it would have been useful to include the option to request the final study results in the online consent materials.

Over half of the participants in our survey indicated a preference for online consent with only three participants indicating a preference for face-to-face consent. Qualitative responses demonstrate that participants found online consent ‘easier’, ‘practical’ and ‘efficient’. These findings would suggest that participants find the online process preferable, but the extent to which it is ‘better’ needs further examination. A crucial part in ascertaining whether the online process is better than a comparable face-to-face procedure would be to define ‘better’. If better is defined in terms of the ability of the online process to mitigate some of the logistical challenges of administering consent, then these findings might add some weight to the argument that online consent is better. For example, many participants noted that had a face-to-face consent process been employed they would not have been able to participate suggesting that, in this study, the convenience of the consent process facilitated recruitment, which consequently increased the statistical power of our serosurvey, and aided generalizability. However, if better is defined in terms of the extent to which valid consent is achieved then with nearly 30 per cent of participants not reading the entire information sheet – and therefore not being in possession of all essential information – these findings suggest that the quality of the consent achieved might be worth reviewing. However, we do not know how this compares to a face-to-face procedure.

The majority of participants in this study indicated a preference for online consent. Some studies argue that participants’ preferences and satisfaction should determine the method of providing informed consent information, suggesting that the most acceptable method for participants constitutes the best approach (Agre *et al.*, 1997; Kraft *et al.*, 2016; Robillard *et al.*, 2017). However, this survey raises questions about the acceptability of online consent and the extent to which this might be an example of the naturalistic fallacy (i.e. just because the online version is ‘desirable’ does not make it better or good) (Salloch *et al.*, 2012). Other researchers have highlighted the tension between patient preferences and ethical requirements (Robillard *et al.*, 2017); however, we could not find any literature drawing normative conclusions about the extent to which patient preferences regarding informed consent ought to be reconciled with the principles and practice of research ethics.

While negative test results were emailed to 261 responders, only 111 (43 per cent) of these completed the consent survey. As the survey was anonymous, there was no way to compare the characteristics of responders and non-responders. It is possible that those who took the time to complete the survey are more amenable to online consent than those who chose not to participate. In addition, for some participants there may have been a considerable delay (up to six months) between consenting to the serostudy and completing the consent survey—this may account for some of the responses indicating that participants had forgotten how much of the consent material they had read.

The study population from which this sample was taken was largely composed of health professionals including: laboratory staff, physicians, nurses and researchers. The survey results, therefore, largely represent the views of individuals familiar with research (and research ethics), as well as the nature of the study. Participants who are themselves involved in carrying out research may have indicated a preference for online consent out of self-interest, as they may see benefits of the approach for their own research.

Though the sample was not typical of research participants, it does provide a valuable survey of health professionals, and speaks to the acceptability of online consent among this population, particularly frontline medical staff. This survey offers valuable information about the potential use of online consent in this population.

## Conclusions

There was a high acceptability of online consent among those who responded. The survey highlights some obvious steps that could be taken to improve the acceptability of online consent. For example, online consent could offer multiple means of communicating with the research team either by including a free-text field at the end of the consent materials, providing the option to request a follow-up call from a member of the research team, or allowing participants to indicate that they would prefer to be consented face-to-face. Further, the option to download or email a copy of the consent materials would likely increase the acceptability of this method.

The majority of participants indicated a preference for online consent. However, participants indicated that the nature of the study lent itself to online consent but that were the study more complicated or risky online consent would not be appropriate. Online consent does not allow researchers to determine if the participant has the capacity to consent, verify the participant’s identity or obtain a signature. Consequently, online consent would not be appropriate as the sole means of consenting participants for clinical trials.^1^ A small number of participants still prefer a face-to-face procedure and where practical, and when this could be done without introducing bias into the study, this should be provided on request.

Finally, it is not possible to understand how online compares to a face-to-face procedure in terms of engagement and comprehension. A comparative study—involving a more typical population of research participants—examining engagement and comprehension is needed. A qualitative study exploring perceptions of voluntariness for both modalities would also provide important insight.

There are few published guidelines for the use of online consent. Guidelines should be developed to detail when online consent is appropriate, what features must be present and how it could best be complimented by other methods. The changing legislative environment governing the processing of personal data in the UK provides a timely opportunity to develop guidelines for the use of online consent.

## Footnotes

1. The legal obligations involved in obtaining consent for clinical trials carried out in the UK are outlined in the Medicines for Human Use (Clinical Trials) Regulations (2004). The use of online consent alone for consenting patients into clinical trials would be unlawful in the UK, and would not meet the Medicines and Healthcare products Regulatory Agency’s Good Clinical Practice standards.

## Appendix 1

Original consent (Page 1)

Original consent (Page 2)

Original consent (Page 3)

Original consent (Page 4)

Original consent (Page 5)

Original consent (Page 6)
